# Prevalence of atherosclerosis and association with 5-year outcome: The Norwegian Stroke in the Young Study

**DOI:** 10.1177/23969873211059472

**Published:** 2021-11-11

**Authors:** Beenish Nawaz, Annette Fromm, Halvor Øygarden, Geir E Eide, Sahrai Saeed, Rudy Meijer, Michiel L Bots, Kristin M Sand, Lars Thomassen, Halvor Næss, Ulrike Waje-Andreassen

**Affiliations:** 1 Department of Clinical Medicine 1, University of Bergen, Bergen, Norway; 2 Department of Neurology, 60498 Haukeland University Hospital, Bergen, Norway; 3 Department of Neurology, Sørlandet Hospital, Kristiansand, Norway; 4 Department of Health and Nursing Sciences, Univeristy of Agder, Kristiansand, Norway; 5 Centre of Clinical Research, 60498 Haukeland University Hospital, Bergen, Norway; 6 Department of Global Public Health and Primary Care, University of Bergen, Bergen, Norway; 7 Department of Cardiology, 60498 Haukeland University Hospital, Bergen, Norway; 8 Julius Center of Health Sciences and Primary Care, 563000 University Medical Center Utrecht, Utrecht University, Utrecht, The Netherlands; 9 Department of Medicine, Sørlandet Hospital, Flekkefjord, Norway; 10 The Institute of Health and Society, Faculty of Medicine, University of Oslo, Oslo, Norway; 11 SESAM, Centre for Age-related Medicine, Stavanger University Hospital, Stavanger, Norway

**Keywords:** Young ischaemic stroke, atherosclerosis, carotid intima-media thickness, femoral intima-media thickness, ankle–arm index, abdominal aorta plaques, cardiovascular events, mortality, long-term outcome, Trial of Org 10172 in Acute Stroke Treatment (TOAST)

## Abstract

**Objectives:**

We studied the prevalence of atherosclerosis among ischaemic stroke patients ≤60 years and controls at the time of the index stroke, and its association with occurrence of new cardiovascular events (CVEs) and mortality at a 5-year follow-up.

**Methods:**

Prevalent atherosclerosis was assessed for 385 patients and 260 controls in seven vascular areas by electrocardiogram (ECG), ankle–arm index (AAI) and measurement of right and left carotid and femoral intima-media thickness (cIMT and fIMT) and abdominal aorta plaques (AAP). Clinical end-points were any new CVE (stroke, angina, myocardial infarction or peripheral arterial disease) or death from any cause at 5-year follow-up. All results were sex- and age-adjusted; logistic regression and Cox proportional hazards models were applied.

**Results:**

Young patients ≤49 years had prevalent atherosclerosis in 1/2 of males and 1/3 of females. Compared with controls, young female patients showed significantly higher prevalent atherosclerosis, *p* = 0.024. Ischaemic ECG and mean cIMT were higher in young and middle-aged female patients (*p* = 0.044, *p* = 0.020, *p* = 0.023 and *p* <0.001, respectively). Mean fIMT was higher in middle-aged female patients (*p* <0.001). Cardiovascular events were associated with ischaemic ECG; AAI ≤0.9, fIMT ≥0.9 mm and increased number of areas with atherosclerosis (NAA) among patients, and with AAP, cIMT ≥0.9 mm, fIMT ≥0.9 mm and NAA among controls. Mortality was associated with higher age, ischaemic ECG and NAA among patients, and cIMT ≥0.9 mm among controls.

**Conclusion:**

Atherosclerosis is highly prevalent even in young stroke patients. Some areas and increasing NAA are associated with CVEs and death.

## Introduction

The 15 cities young stroke study showed smoking, dyslipidemia and hypertension as the three most frequent risk factors (RF) for cardiovascular events (CVEs), without regional differences in Europe.^1^ Several European long-term young stroke studies have shown high rates of recurrent CVEs, such as ischaemic stroke (IS), angina, myocardial infarction (MI), peripheral arterial disease (PAD) and mortality mainly due to coronary atherosclerosis (CA).^2,3^ Autopsy studies have also shown high prevalence of CA, predominantly in young healthy male populations.^4,5^

The 15 cities study found 39.6% stroke of undetermined cause (SUC) and 9.3% large-artery atherosclerosis (LAA) based on the Trial of Org 10172 in Acute Stroke Treatment (TOAST) classification, requiring ≥50% artery stenosis.^6^

Combining knowledge of the high rates of recurrent CVEs and mortality in young stroke patients,^7^ worst prognosis for patients with atherosclerosis,^8^ high rates of cryptogenic stroke among young patients,^9^ and knowledge showing that plaque instability is more important than the degree of stenosis for coronary and cerebral CVEs,^10,11^ we wanted to investigate the extent of atherosclerosis in young stroke patients. Staging of arterial vascular areas, with a more scrutineous detection of atherosclerosis, became a main pillar for the Norwegian Stroke in the Young Study (NOR-SYS). Our hypothesis is that atherosclerosis is far more present among young and middle-aged patients than the TOAST definition of atherosclerosis with at least 50% stenosis^12^ is able to show, and arterial staging is the first step to understand the extent of established artery wall disease. The aims of this study are the detailed presentation of seven predefined vascular areas at study inclusion, and their association with new CVEs and mortality at 5-year follow-up.

## Methods

### Participants

From 1st September 2010 to 31st December 2015, 385 stroke patients aged 15–60 years, and 260 partners, serving as controls aged 21–69 years, were included. NOR-SYS design and ultrasound protocol,^13^ and methods and results of inclusion have been published.^14^ Verified acute ischaemic stroke diagnosis was done by magnetic resonance imaging (MRI) in 98.5% among patients or by computed tomography (CT) alone in case of contraindications. The majority of stroke patients were admitted within a few days after symptom onset (Table 1). However, in some cases, inclusion in NOR-SYS was delayed, often when patients got the stroke at other sites in Norway or abroad.

**Table 1.** Overview over time of admission to our hospital from acute stroke symptom onset and inclusion into the Norwegian Stroke in the Young Study.

Time of admission	Patients, *n* (%)
<24 h (h)	256 (66.5)
≥24 h to <72 h	59 (15.3)
≥72 h to <1 week	21 (5.4)
≥1 week to <1 month	27 (7.0)
≥1 month	22 (5.7)

Longest time delay appeared mainly due to first admission to other hospitals in Norway or abroad.

### Baseline procedures

A 12-lead electrocardiogram (ECG) was verified by a cardiologist to identify signs of acute or previous myocardial ischaemia. Detailed information about the NOR-SYS ultrasound protocol has been published before.^13^ In brief, ankle–arm index (AAI) ≤0.9 indicated PAD. High quality measurements of mean carotid intima-media thickness (cIMT) and femoral intima-media thickness (fIMT) were obtained at predefined segments from the common carotid artery, carotid bifurcation, internal carotid artery, common femoral artery and superficial femoral artery. In the analyses, the maximum of any mean IMT segment value was used, and plaques were included in the IMT measurements. Mean IMT values ≥0.9 mm were considered pathological,^15^ and mean IMT values ≥1.5 mm were defined as atherosclerosis.^16^ The abdominal aorta was assessed by ultrasound for detection of abdominal aorta plaques (AAP).

### Prevalent atherosclerosis

Atherosclerosis was defined prevalent at seven chosen areas by the following: presence of ischaemic ECG signs; AAI ≤0.9; right and left mean cIMT and fIMT ≥ 1.5 mm, respectively, and presence of AAP. Presence of atherosclerosis on each vascular area was assigned a value of 1. Atherosclerosis was defined as number of affected vascular areas 0–7.

### Stroke classification

Stroke aetiology was classified according to TOAST criteria, independent from the results of the ultrasound research protocol.

### Follow-up data

Patients and controls visited our outpatient clinic from 1^st^ September 2015 to 31^st^ December 2020 for a 5-year follow-up. Primary end-points were occurrence of any new CVE (ischaemic or haemorrhagic stroke; angina or MI; and PAD), and death of any cause. Study participants were interviewed about occurrence of any CVE during the follow-up period, and new CVEs were verified by medical records for those who attended the follow-up. ECG were performed. For mortality analysis, we chose the date of 31st August 2020. In Norway, mortality data appears in medical records, connected to each citizen’s 11-digit personal identification number.

### Ethical considerations

The study complies with the Declaration of Helsinki and is approved by the Regional Ethics Committee (REK-Vest 2010/74). Written consent is present for all study participants. The Regional Ethics Committee did not allow to follow dropouts apart from the dead–alive state and causes of death.

### Statistics

The mean and standard deviation (SD) were used for descriptive statistics. Study participants were dichotomised into young (≤49 years) and middle-aged (≥50 years). Analyses were adjusted by age and sex. To avoid systematic bias, univariate comparisons of vascular areas between patients and controls were done within the four sex and age strata using the unpaired t-test and the Fisher’s exact test. For the unadjusted comparison of all patients to their controls, McNemar’s test of symmetry was used. Mean cIMT and fIMT values were compared between patients and controls by relative change (RC). The number of vascular areas affected by atherosclerosis was compared between TOAST subgroups using the Kruskal–Wallis rank test. To adjust for confounding and matching, logistic regression and Cox models were used to estimate the risk of new CVE and mortality, with respect to age, sex and vascular areas. The results were reported as odds ratios (OR) and hazard ratios (HR) with a 95% confidence interval (CI). Two-sided *p*-values ≤0.05 were considered significant. All statistical analyses were performed in Stata SE 16.0.

## Results

### Study population

At inclusion, patients had a mean age of 49.5 years, and controls had a mean age of 50.3 years (Table 2). The majority of patients were males (68.6%), and the majority of controls were females (70.0%). Young age ≤49 years were present for 39.5% of patients and 39.2% of controls.

**Table 2.** Baseline characteristics of patients and controls at inclusion and at 5-year follow-up.

		NA *n*	All	Male ≤49 years	Male ≥50 years	Female ≤49 years	Female ≥50 years
At inclusion							
Patients, *n* (%)	P		385 (100.0)	94 (35.6)	170 (64.4)	58 (47.9)	63 (52.1)
Controls, *n* (%)	C		260 (100.0)	28 (35.9)	50 (64.1)	74 (40.7)	108 (59.3)
Age (y), *mean (SD*)	P	0	49.5 (9.8)	40.5 (8.2)	55.9 (3.0)	38.4 (8.9)	55.8 (2.9)
	C	0	50.3 (8.6)	42.0 (6.7)	57.5 (4.9)	41.7 (6.6)	55.0 (3.2)
Unpaired T-test, p^a^			0.001*	0.312	**0.028**	**0.019**	0.102
Ischaemic ECG, *n* (%)	P	0	40 (10.4)	9 (9.6)	17 (10.0)	6 (10.3)	8 (12.7)
	C	4	7 (2.7)	1 (3.6)	2 (4.1)	1 (1.4)	3 (2.8)
Fisher’s exact test, *p*			0.002**	0.450	0.259	**0.044**	**0.020**
AAI ≤0.9, *n (%)*	P	23	17 (4.7)	2 (2.3)	9 (5.7)	2 (3.6)	4 (6.8)
	C	5	7 (2.7)	1 (3.7)	3 (6.3)	1 (1.4)	2 (1.9)
Fisher’s exact test, *p*			1.000**	0.559	1.000	0.577	0.188
AAP, *n (%)*	P	39	162 (46.8)	29 (33.0)	90 (58.8)	13 (25.0)	30 (56.6)
	C	13	87 (35.2)	5 (18.5)	29 (61.7)	9 (12.7)	44 (43.1)
Fisher’s exact test, *p*			0.002**	0.158	0.865	0.097	0.129
At 5-year follow-up							
Patients, *n* (%)	P	62	323 (83.9)	82 (36.1)	145 (63.9)	47 (49.0)	49 (51.0)
Controls, *n* (%)	C	41	219 (84.2)	21 (35.6)	38 (64.4)	64 (40.0)	96 (60.0)
Deceased, *n* (%)	P	0	21 (5.5)	1 (1.1)	13 (7.6)	2 (3.4)	5 (7.9)
	C	0	9 (3.5)	0 (0.0)	5 (10.0)	1 (1.4)	3 (2.8)
Fisher’s exact test, *p*			0.524**	1.000	0.560	0.582	0.146
Any CVE, *n* (%)	P	2	44 (13.7)	10 (12.3)	22 (15.3)	7 (14.9)	5 (10.2)
	C	0	9 (4.1)	1 (4.8)	2 (5.3)	0 (0.0)	6 (6.3)
Fisher’s exact test, *p*			0.001**	0.451	0.172	**0.002**	0.509
Stroke^b^, *n* (%)	P	2	26 (8.1)	7 (8.6)	10 (6.9)	6 (12.8)	3 (6.3)
	C	0	0 (0.0)	0 (0.0)	0 (0.0)	0 (0.0)	0 (0.0)
Fisher’s exact test, *p*			< 0.001**	0.340	0.218	**0.005**	**0.037**
Angina and/or MI, *n* (%)	P	2	16 (5.0)	5 (6.1)	8 (5.6)	2 (4.3)	1 (2.04)
	C	0	8 (3.6)	1 (4.8)	2 (5.1)	0 (0.0)	5 (5.2)
Fisher’s exact test, *p*			0.002**	1.000	1.000	0.177	0.664
PAD, *n* (%)	P	1	11 (3.4)	1 (1.2)	8 (5.6)	1 (2.1)	1 (2.1)
	C	0	1 (0.4)	0 (0.0)	0 (0.0)	0 (0.0)	1 (1.0)
Fisher’s exact test, *p*			0.07**	1.000	0.363	0.423	1.000

Abbreviations: NA: not available, missing data; *n*: number of subjects; y: years; P: patients; C: controls; SD: standard deviation; ECG: electrocardiogram; AAI: ankle–arm index; AAP: abdominal aorta plaques; MI: myocardial infarction; PAD: peripheral artery disease.

*Paired T-test; ** Exact McNemar’s test of symmetry.

^a^*p*-values are comparisons between patients and controls.

^b^Stroke included haemorrhagic and ischaemic stroke. Two patients had haemorrhagic stroke.

### Clinical and ultrasonographic findings

Compared to controls, young aged and middle-aged female patients had higher prevalence of ischaemic ECG (*p* = 0.044 and *p* = 0.020) and higher mean cIMT (*p* = 0.023 and *p* < 0.001), as shown in Tables 2 and 3. Mean fIMT was higher in middle-aged female patients (*p* <0.001). Abdominal aorta plaques presence and pathological AAI did not differ in any comparisons between patients and controls. The prevalence of atherosclerosis among male patients and controls did not differ for clinical and ultrasonographic variables.

**Table 3.** Ultrasound protocol on mean IMT^a^ from carotid and femoral arteries of patients and controls.

		NA *n*	All	Male ≤49 years	Male ≥50 years	Female ≤49 years	Female ≥50 years
Patients, *n* (%)			385 (100.0)	94 (35.6)	170 (64.4)	58 (47.9)	63 (52.1)
Controls, *n* (%)			260 (100.0)	28 (36.4)	50 (64.1)	74 (40.4)	108 (59.3)
Carotid arteries, cIMT							
Relative change % (95% CI)			22 (13, 31)	6 (−16, 30)	10 (−8, 28)	17 (2, 32)	31 (16, 46)
Mean cIMT (SD)	P	2	1.35 (0.90)	1.02 (0.57)	1.60 (0.94)	0.90 (0.55)	1.59 (1.13)
	C	1	1.05 (0.52)	0.95 (0.37)	1.44 (0.71)	0.74 (0.17)	1.10 (0.47)
Unpaired t-test, *p*			<0.001**	0.578	0.278	**0.023**	**<0.001**
CCA							
Relative change % (95% CI)			10 (5, 15)	2 (−10, 13)	2 (−8, 11)	2 (−4, 8)	15 (6, 24)
Mean CCA-IMT (SD)	P	2	0.84 (0.29)	0.74 (0.21)	0.94 (0.30)	0.64 (0.14)	0.90 (0.34)
	C	1	0.75 (0.21)	0.73 (0.14)	0.92 (0.27)	0.62 (0.09)	0.77 (0.19)
Unpaired t-test, *p*			<0.001**	0.777	0.758	0.485	**0.001**
BIF							
Relative % change, 95% CI			18 (10, 26)	1 (−18, 20)	8 (−9, 24)	12 (−14, 25)	26 (11, 42)
Mean BIF-IMT (SD)	P	3	1.19 (0.73)	0.90 (0.38)	1.41 (0.75)	0.79 (0.40)	1.42 (0.96)
	C	1	0.98 (0.46)	0.90 (0.35)	1.30 (0.54)	0.70 (0.17)	1.04 (0.46)
Unpaired t-test, *p*			<0.001**	0.945	0.345	0.079	**<0.001**
ICA							
Relative % change, 95% CI			25 (14,−35)	9 (−18, 36)	12 (−12, 35)	18 (1, 36)	35 (17, 53)
ICA-IMT (SD)	P	9	1.00 (0.79)	0.81 (0.56)	1.15 (0.85)	0.70 (0.51)	1.17 (0.99)
	C	3	0.75 (0.44)	0.73 (0.26)	1.02 (0.73)	0.57 (0.15)	0.77 (0.37)
Unpaired t-test, *p*			<0.001**	0.506	0.329	**0.042**	**<0.001**
Femoral arteries, fIMT							
Relative % change, 95% CI			31 (21, 42)	28 (−5, 60)	14 (−6, 34)	15 (−6, 37)	31 (13, 49)
Mean fIMT (SD)	P	7	1.42 (1.08)	1.13 (0.92)	1.82 (1.19)	0.75 (0.51)	1.42 (0.96)
	C	1	0.98 (0.76)	0.82 (0.47)	1.57 (0.99)	0.64 (0.44)	0.97 (0.73)
Unpaired t-test, *p*			<0.001**	0.095	0.176	0.164	**<0.001**
CFA							
Relative % change, 95% CI			31 (20, 42)	26 (−6, 58)	14 (−6, 34)	15 (7, 37)	31 (13, 49)
Mean CFA-IMT (SD)	P	7	1.41 (1.08)	1.11 (0.90)	1.81 (1.19)	0.75 (0.51)	1.41 (0.96)
	C	1	0.97 (0.77)	0.82 (0.47)	1.56 (0.99)	0.64 (0.44)	0.97 (0.73)
Unpaired t-test, *p*			<0.001**	0.115	0.170	0.169	**0.001**
SFA							
Relative % change, 95% CI			20 (10, 30)	15 (−11, 41)	17 (−8, 43)	12 (2, 22)	10 (−1, 21)
Mean SFA-IMT (SD)	P	8	0.60 (0.47)	0.56 (0.37)	0.70 (0.62)	0.47 (0.18)	0.54 (0.13)
	C	2	0.48 (0.19)	0.47 (0.13)	0.58 (0.22)	0.42 (0.07)	0.49 (0.22)
Unpaired t-test, *p*			<0.001**	0.254	0.184	**0.014**	0.072

Abbreviations: NA: not available, missing data; *n*: number of subjects; y: years; cIMT/fIMT/IMT: carotid/femoral intima-media thickness; CI: confidence interval; SD: standard deviation; P: patients; C: controls; CCA: common carotid artery; BIF: carotid bifurcature; ICA: internal carotid artery; CFA: common femoral artery; SFA: superficial femoral artery.

**Paired t-test.

^a^Mean IMT measurements units were millimetres, and were obtained bilaterally from 1 cm segment at four standardised angles for CCA using Meijer’s Carotid Arc® and at one angle for BIF, ICA, CFA and SFA. Among several measurements at any segmental level, the maximal IMT value was used.

Eight (5.1%) of 157 patients with fIMT ≥0.9 mm in their right femoral artery (FA) had previously performed percutaneous coronary intervention (PCI) with access from their right FA.

### Missing data

Ultrasonography was not performed in two patients due to terminal unconsciousness at admission and morbid obesity causing insufficient imaging quality, respectively. Main reasons for other missing data (Table 3) were arterial occlusion due to atherosclerosis or dissection, bad imaging quality, air (AAP) or anatomical reasons. Good quality measurements of at least 70% segmental analysis for cIMT and fIMT were averagely achieved in 96.7%.

### Prevalence of atherosclerosis

**Figure 1.**
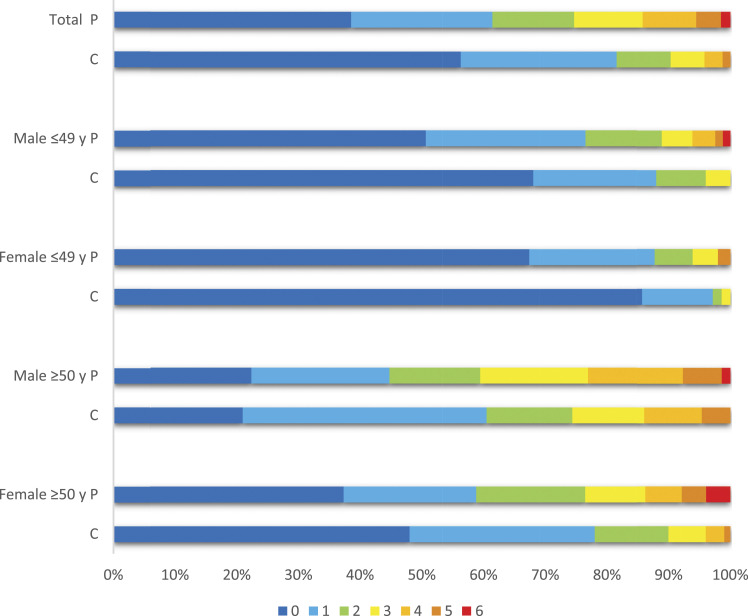
Prevalence of atherosclerosis at different vascular areas^a^ among 324 stroke patients and 238 controls. Abbreviations: P: patients; C: controls; y: years. (a) Atherosclerosis was evaluated in seven vascular areas by electrocardiogram, ankle–arm index and by ultrasonography of abdominal aorta and right and left carotid and femoral arteries for intima-media thickness (IMT) measurements. Among several measurements at any segmental level, the maximum IMT value was used.

The prevalence of atherosclerosis was higher in young female patients (32.7% vs 14.3%, *p* = 0.024) compared with young female controls (Figure 1). The prevalence did not differ between patients and controls among young male patients (49.4% vs 32.0%, *p* = 0.167), middle-aged male patients (77.6% vs 78.6%, *p* = 1.000) and middle-aged female patients (62.7% vs 52.5%, *p* = 0.229). The prevalence was higher in middle-aged patients than in younger patients (male 77.6% vs 49.4%, *p* <0.001, female 62.7% vs 32.7%, *p* = 0.026) and higher in male patients than in female patients (67.4% vs 48.0%, *p* = 0.014). Maximum number of affected vascular areas were six among patients and five among controls.

### Stroke classification

**Figure 2.**
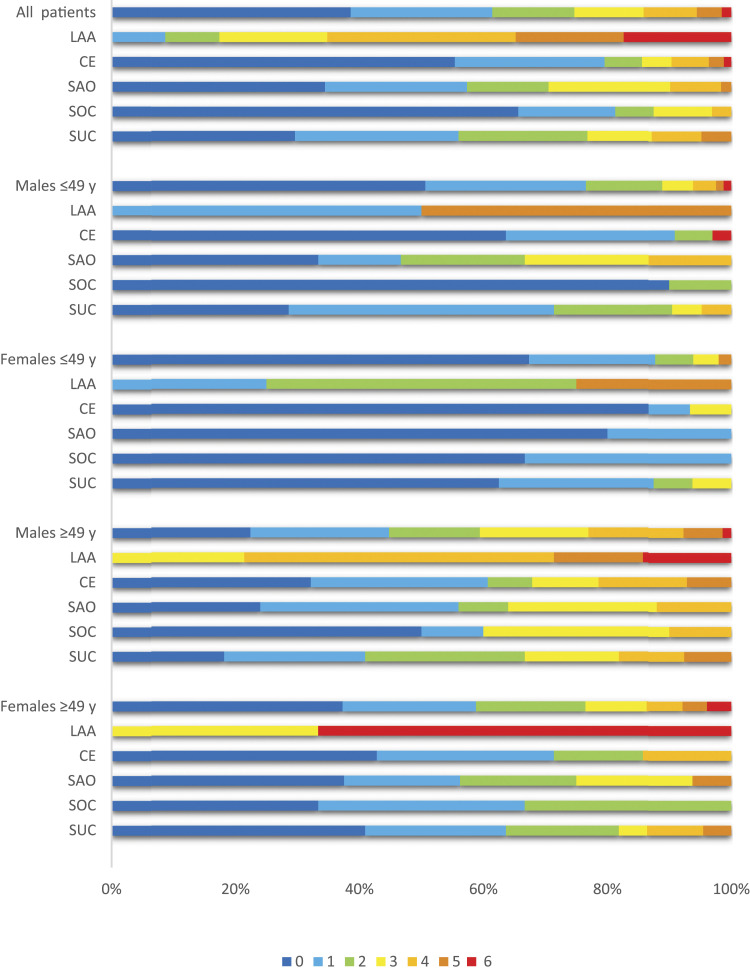
Prevalence of atherosclerosis at different vascular areas^a^ among 324 stroke patients related to TOAST classification. Abbreviations: TOAST: Trial of Org 10172 in Acute Stroke Treatment; LAA: large-artery atherosclerosis; CE: cardiac embolism; SAO: small artery occlusion; SOC: stroke of other determined cause; SUC: stroke of undetermined cause; y: years. (a) Atherosclerosis was evaluated in seven vascular areas by electrocardiogram, ankle–arm index and by ultrasonography of abdominal aorta and right and left carotid and femoral arteries for intima-media thickness (IMT) measurements. Among several measurements at any segmental level, the maximum IMT value was used.

LAA was found among 28 (7.3%) of patients according to the TOAST classification (Table 4). Atherosclerosis was present among all of the patients in LAA group, and least present by 34.4% among patients of stroke with other determined cause (SOC) (*p* <0.001), Figure 2. In the SUC group ≤49 years, 71.4% of males and 37.5% of females had prevalent atherosclerosis.

**Table 4.** Stroke subtypes according to TOAST classification in young stroke patients related to gender and age group.

	All *n* = 385	Men		Women			
Stroke subtypes, *n* (%)		≤49 years *n* = 94	≥50 years *n* = 170	≤49 years *n* = 58	≥50 years *n*= 63	*p*-value sex	*p*-value age group
LAA	28 (7.3)	2 (2.1)	17 (10.0)	4 (6.9)	5 (7.9)		
CE	100 (26.0)	36 (38.3)	40 (23.3)	17 (29.3)	7 (11.1)		
SAO	73 (19.0)	17 (18.1)	29 (17.1)	6 (10.3)	21 (33.3)	0.385	<0.001
SOC	41 (10.6)	16 (17.0)	10 (5.9)	11 (19.0)	4 (6.3)		
SUC	143 (37.1)	23 (24.5)	74 (43.5)	20 (34.5)	26 (41.3)		

Abbreviations: TOAST: Trial of Org 10172 in Acute Stroke Treatment; LAA: large-artery atherosclerosis; CE: cardiac embolism; SAO: small artery occlusion; SOC: stroke of other determined cause; SUC: stroke of undetermined cause; *p*-value: from Chi-square test.

### Follow-up data

The average duration of our outpatient clinical follow-up was 5.3 years for all participants. There were 323 (83.9%) patients and 219 (84.2%) controls participating in the 5-year follow-up. Three patients had telephone interviews. During follow-up, 44 patients (13.7%) and 9 controls (4.1%) experienced any CVE (*p* = 0.001), and occurrence of CVE was higher in young female patients than controls (*p* = 0.002). In total, 21 (5.5%) patients and 9 (3.5%) controls died (*p* = 0.524). No difference was found after adjustment for age and sex. Seven patients died within 1 month after hospital admission due to malign oedema (4), basilarisocclusion (2) and subarachnoidal haemorrhage (1). Regarding 14 patients who died after the first month after hospital admission, and within 31th August 2020, when dead–alive state was checked, the causes of deaths were recurrent stroke (1), coronary heart disease (1), lung embolism (1), cancer (4), infection (3), dementia (1) and unknown cause (3). The causes of death among controls were cancer (4), infection (1), respiratory failure after lung transplant (1) and unknown cause (3).

### CVEs and mortality

Adjusted for sex and age, occurrence of CVEs was associated with ischaemic ECG (OR 3.48; *p* = 0.005), AAI ≤0.9 (OR 5.15; *p* = 0.004), fIMT ≥0.9 mm (OR 2.48; *p* = 0.019) and increased number of areas with atherosclerosis (NAA) (OR 1.31; *p* = 0.025) among patients, and with presence of AAP (OR 7.41; *p* = 0.023), cIMT ≥0.9 mm (OR 11.17; *p* = 0.042), fIMT ≥0.9 mm (OR 9.69; *p* = 0.010) and increased NAA (OR 1.81; *p* = 0.014) among controls (Table 5). Adjusted for all variables, we did not find any significant results with occurrence of CVEs. No interactions were found between sex, age and vascular areas. Adjusted for sex and age, mortality was associated with higher age (HR 1.08; *p* = 0.036), ischaemic ECG (HR 3.51; *p* = 0.009) and increased NAA (HR 1.36; *p* = 0.047) among patients, and with cIMT ≥0.9 mm (HR 8.26; *p* = 0.013) among controls (Table 6). Adjusted for all variables, mortality was associated with increased NAA (HR 1.98; *p* = 0.047) among patients, and with cIMT ≥0.9 mm (HR 8.51; *p* = 0.014) among controls.

**Table 5.** Results from logistic regression of any new cardiovascular event within 5-year follow-up of 323 patients and 219 controls.

Groups		Adjusted for sex and age			Adjusted for all variables		
Variables	*n*	OR	95% CI	*p*-value	OR	95% CI	*p*-value
Patients						*n* = 273	
Male sex	321	0.93	(0.45, 1.92)	0.851	1.30	(0.55, 3.08)	0.544
Age at inclusion (y)	321	1.03	(0.99, 1.07)	0.125	1.02	(0.97, 1.07)	0.486
Ischaemic ECG	321	3.48	(1.45, 8.33)	**0.005**	2.28	(0.61, 8.44)	0.218
AAI ≤0.9	309	5.15	(1.68, 15.84)	**0.004**	3.29	(0.79, 13.71)	0.103
AAP	288	1.45	(0.70, 3.02)	0.318	0.96	(0.33, 2.80)	0.947
cIMT ≥0.9 mm	321	1.82	(0.80, 4.14)	0.155	0.92	(0.36, 2.39)	0.869
fIMT ≥0.9 mm	317	2.48	(1.16, 5.30)	**0.019**	2.22	(0.79, 6.20)	0.129
NAA^a^	260	1.31	(0.00, 0.32)	**0.025**	1.09	(0.73, 1.63)	0.685
Controls						***n* = 197**	
Male sex	219	0.82	(0.19, 3.49)	0.789	1.80	(0.37, 8.71)	0.465
Age at inclusion (y)	219	1.05	(0.95, 1.16)	0.311	0.95	(0.84, 1.09)	0.473
Ischaemic ECG	210	0.00	—	—	0.00	—	—
AAI ≤0.9	216	3.81	(0.36, 40.05)	0.265	2.07	(0.09, 47.96)	0.650
AAP	208	7.41	(1.32, 41.59)	**0.023**	3.37	(0.39, 29.21)	0.270
cIMT ≥0.9 mm	218	11.17	(0.00, 6.30)	**0.042**	5.53	(0.48, 63.30)	0.169
fIMT ≥0.9 mm	218	9.69	(1.71, 55.03)	**0.010**	4.16	(0.62, 27.97)	0.143
NAA^a^	202	1.81	(1.13, 2.91)	**0.014**	1.05	(0.48, 2.33)	0.898

Abbreviations: n: number of subjects; OR: odds ratio; CI: confidence interval; y: years; ECG: electrocardiogram; AAI: ankle–arm index; AAP: abdominal aorta plaque; cIMT/fIMT: carotid/femoral intima-media thickness.

^a^NAA **=** number of vascular areas with atherosclerosis, indicating whether there is increasing trend with increasing NAA.

**Table 6.** Results of Cox regression of mortality risk within 5-year follow-up of 385 patients and 260 controls.

Groups		Adjusted for sex and age			Adjusted for all variables		
Variables	*n*	HR	95% CI	*p*-value	HR	95% CI	*p*-value
Patients						*n* = 323	
Male sex	383	1.24	(0.50, 3.09)	0.642	1.51	(0.47, 4.84)	0.487
Age at inclusion (y)	383	1.08	(1.00, 1.15)	**0.036**	1.09	(0.99, 1.19)	0.081
Ischaemic ECG	383	3.51	(1.36, 9.07)	**0.009**	1.52	(0.33, 7.04)	0.596
AAI ≤0.9	360	2.90	(0.64, 13.09)	0.165	0.77	(0.12, 5.13)	0.786
AAP	345	1.08	(0.41, 2.81)	0.880	0.35	(0.06, 1.94)	0.231
cIMT ≥0.9 mm	381	0.59	(0.23, 1.52)	0.271	0.36	(0.08, 1.56)	0.171
fIMT ≥0.9 mm	376	0.85	(0.33, 2.19)	0.734	0.59	(0.11, 3.10)	0.533
NAA^a^	310	1.36	(1.00, 1.84)	**0.047**	1.98	(1.01, 3.86)	**0.047**
Controls						***n* = 219**	
Male sex	239	2.84	(0.61, 13.21)	0.183	3.42	(0.73, 16.02)	0.119
Age at inclusion (y)	239	1.07	(0.98, 1.16)	0.132	1.01	(0.92, 1.12)	0.776
Ischaemic ECG	235	3.36	(0.44, 25.98)	0.245	2.67	(0.23, 31.55)	0.436
AAI ≤0.9	235	0.00	(0,−)	1.000	0.00	—	—
AAP	227	0.98	(0.30, 3.20)	0.972	0.76	(0.12, 4.88)	0.776
cIMT ≥0.9 mm	238	8.26	(1.57, 43.41)	**0.013**	8.51	(1.55, 46.72)	**0.014**
fIMT ≥0.9 mm	238	1.09	(0.32, 3.74)	0.886	0.60	(0.13, 2.74)	0.507
NAA^a^	219	1.14	(0.71, 1.83)	0.578	1.13	(0.48, 2.69)	0.776

Abbreviations: n: number of subjects; HR: hazards ratio; CI: confidence interval; y: years; ECG: electrocardiogram; AAI: ankle–arm index; AAP: abdominal aorta plaque; cIMT/fIMT: carotid/femoral intima-media thickness.

^a^NAA: number of vascular areas with atherosclerosis, indicating whether there is increasing trend with increasing NAA.

## Discussion

### Prevalent atherosclerosis

To our knowledge, this is the first study of young and middle-aged acute IS patients and controls which has described the state of the arteries at different vascular areas, regardless of the cause of stroke. The overall result is that atherosclerosis is prevalent even in young patients and controls, and middle-aged males are most affected, confirming established knowledge. Detailed assessment demonstrated atherosclerosis in half of young male patients and one third of young female patients. Young patients had numerically more atherosclerosis than controls had, but there was a statistical difference only between young female patients and controls.

Our findings are in line with other studies showing that clinical CVEs are only ‘the tip of the iceberg’,^17^ and that subclinical and clinical atherosclerosis starts in early life, and increases with age, particularly in males.^2^ The Aragon Workers’ Health Study assessed 1423 males, aged 40–59 years, for coronary artery calcium score and carotid and femoral plaques, and reported subclinical atherosclerosis in 72% of participants.^18^ Hormonal influences are assumed to contribute to the delayed development of atherosclerosis in females.^19^

### Surrogate markers for atherosclerosis

Since atherosclerosis is regarded as a risk factor for CVEs and mortality, various non-invasive surrogate markers for atherosclerosis have been identified.^20^ We found that all of the investigated vascular areas were associated with CVEs in patients as well as controls. We found that increased fIMT was common vascular area among patients and controls that predicted CVEs. Previous studies have associated fIMT with extent of coronary atherosclerosis^21^ and carotid atherosclerosis,^22^ and regarded fIMT as a surrogate marker for atherosclerosis.^23^ Kocygit et al. followed 215 subjects (mean age 54.85 years) for a median of 24 months and found that femoral plaques were independent predictors for CVEs.^24^ Giannoukas et al. reported that fIMT separately or in combination with cIMT was related to cardiovascular disease.^25^

CIMT and AAI are well-established surrogate markers for subclinical atherosclerosis and strong predictors of future CVEs and mortality.^20,26,27^ In the ARIC (Atherosclerosis risk in communities) study, cIMT predicted CVEs or death in participants recruited from four communities in the United States.^27^ A meta-analysis has shown that increased cIMT by 0.10 mm is associated with an increased risk of 18% for stroke and 15% for myocardial infarction.^26^ Our study confirms that cIMT predicts increased risk of CVEs and mortality among controls and that AAI is strongly related to CVE among patients.

Another important finding in our study is that ischaemic ECG predicts CVEs and mortality among patients, regardless of age and sex. Also, Bacquer et al. reported that major abnormalities in ECG are strongly associated with CVE and mortality in both sexes.^28^ Furthermore, ECG findings revealing silent ischaemia has been a powerful and independent predictor for cardiac mortality in another study.^29^

Regarding AAP, we found a positive association with CVEs among controls. Li et al. reported higher prevalence of AAP in patients with coronary artery disease (CAD), compared to those without CAD.^30^ In the Rotterdam study, AAP, measured by X-ray in 6389 subjects, was associated with MI.^31^

### Stroke classification by TOAST

The TOAST classification is most widely used by date, but there is the problem of the big group of up to 33–40% of young stroke patients with SUC,^6,32^ and this is in line with our results.

### Strengths and limitations

The major strength of NOR-SYS is the population-based design with inclusion of consecutive acute IS patients and comprehensive vascular work-up based on a standardised protocol. The number of unobtainable IMT measurements was low. We used mean IMT measurements, which provide information on cardiovascular risk even in absence of plaques.^33^ PCI of the FA is associated with haematoma, intimal dissection or arterial occlusion.^34^ However, in our study, only few patients with increased fIMT on the right side had undergone PCI.

An important study limitation was inequality of sex group sizes, with a high number of male patients and relatively low number of male controls. Our controls were partners of included patients, selected as such to improve the three-generation design of NOR-SYS, and also including joint offspring. We expected higher risk factor matching between patients and partners compared with controls selected by random.^35^ We chose not to include intracranial arterial pathology analysis due to uncertainties in defining the cause and degree of stenosis by common imaging methods.^36^ Another limitation was the low number of outcome events, especially numbers of deaths.

## Conclusion

Atherosclerosis is highly prevalent in our study population, being found in half of the young male patients and one third of the young female patients ≤49 years. Comprehensive investigation reveals a far higher prevalence of atherosclerosis than found by TOAST criteria.

In oncology, staging of tumours has for decades been the first step to tailor individual treatment. Staging arteries of young stroke patients should be performed, accordingly. This would contribute to potential early individual-tailored secondary prophylaxis, and selection of patients to targeted and reasonable treatment, and future more targeted genetic diagnostics in order to search for why we do find serious early arterial disease (LAA) in some patients, while other patients are protected from development of atherosclerosis despite of a high number of risk factors. Ultrasound images of good quality were used in this study as teaching tool to explain interested patients the findings and importance of life-style changes, and to take and continue medication that we evaluate necessary for secondary prevention. Randomised future studies could show if this could contribute significantly to reach important treatment goals.
